# Exploring barriers to accessing treatment for Lymphatic Filariasis through a socio-ecological lens in Buvuma and Napak districts, Uganda

**DOI:** 10.1371/journal.pntd.0012747

**Published:** 2025-01-09

**Authors:** Arthur Bagonza, Linda Gibson, Lydia Kabiri, Chris Bezalel, Saravu Rama Bhat Narahari, Peter J. Franks, David Musoke, Christine Moffatt

**Affiliations:** 1 Department of Community Health and Behavioural Sciences, Makerere University College of Health Sciences, School of Public Health, Kampala, Uganda; 2 Institute of Health & Allied Professions, School of Social Sciences, Nottingham Trent University, Nottingham, United Kingdom; 3 Department of Nursing, Makerere University College of Health Sciences, School of Health Sciences, Kampala, Uganda; 4 Institute of Applied Dermatology, Kasaragod, Kerala, India; 5 International Lymphoedema Framework, London, United Kingdom; 6 Centre for Research & Implementation of Clinical Practice, London, United Kingdom; 7 Department of Disease Control and Environmental Health, Makerere University College of Health Sciences, School of Public Health, Kampala, Uganda; 8 Nottingham University Hospitals, NHS Trust Nottingham, Nottingham, United Kingdom; Federal University of Agriculture Abeokuta, NIGERIA

## Abstract

**Background:**

The World Health Organization launched the Global Programme to Eliminate Lymphatic Filariasis in 2000, which aimed at eradicating the disease by 2030. This goal depends on community mass drug administration and essential care. Despite these efforts, many rural communities still face untreated lymphatic filariasis and lack access to treatment and self-management. Using a socioecological model, this study examined barriers to treatment and support for people living with lymphatic filariasis in Uganda’s Buvuma and Napak districts.

**Methods:**

We conducted 12 key informant interviews with health facility officials, district health officers, and village health team members. Additionally, 19 focus group discussions were held with community members, and nine in-depth interviews were conducted with people living with lymphatic filariasis. This study explored knowledge gaps among those living with lymphatic filariasis and health providers that affect morbidity management and disability prevention, as well as challenges in accessing treatment. Audio recordings were transcribed and managed via ATLAS ti software version 6. Thematic analysis was conducted via the socioecological model framework.

**Results:**

This study identified multiple complex factors affecting healthcare providers and individuals living with lymphatic filariasis, spanning individual, interpersonal, organisational, community, and policy levels. The key themes included stigma and social isolation, healthcare disparities, and healthcare dynamics. Stigma and social isolation result in emotional distress, neglect, exclusion, and self-isolation, which are influenced by community beliefs. Health systems and policy barriers included poverty, inadequate personnel, and insufficient equipment. Structural factors such as geographical remoteness, environmental harshness, and lack of healthcare infrastructure were prominent obstacles to seeking care for lymphatic filariasis management.

**Conclusion:**

This study provides insights into the multilevel factors influencing the understanding and availability of treatment for lymphatic filariasis in Uganda. Addressing sociocultural beliefs, social and interpersonal dynamics, and healthcare disparities is crucial for improving the outcomes and well-being of these rural communities. These findings can aid in managing lymphatic filariasis in Uganda and similar low-resource settings.

## Author summary

In many rural parts of Uganda, people living with swelling in their limbs, known as lymphatic filariasis, face significant challenges in receiving the treatment they need. This condition is often caused by infected mosquitoes biting humans and transferring parasitic worms from the Filariodidea family, primarily *Wuchereria bancrofti*, *Brugia malayi*, and *Brugia timori*, into the bloodstream, ending up in the lymphatic system. Despite efforts to eliminate this disease, many people still suffer without proper care.

Our study examined the difficulties these individuals and their healthcare providers encounter in the Buvuma and Napak districts. We talked to health workers and people with lymphatic filariasis to understand their experiences. We found that various factors, such as stigma, social isolation, a lack of healthcare resources, and poor infrastructure, make it difficult for people to obtain the help they need.

These findings are important because they highlight the need for better support and resources for managing lymphatic filariasis. By understanding the barriers to treatment, we can develop better policies and interventions to help those affected. Our research aims at improving health outcomes and quality of life for people with lymphoedema in Uganda and similar areas. The work fits in the broader context of global health by addressing the ongoing challenges of neglected diseases in resource-poor settings.

## Introduction

Cellulitis and lymphatic filariasis are common conditions that burden people affected by these conditions and healthcare systems globally, both in high- and low-income settings [1]. However, these conditions are frequently underrecognized, leading to limited medical system support for the estimated 250 million affected individuals worldwide. Despite their prevalence, there is inadequate access to treatment among people living with lymphatic filariasis and inadequate knowledge of the management of conditions among health workers in many low- and middle-income countries (LMICs) [2]. Furthermore, significant disparities in diagnosis, treatment, and funding exist within and across many countries, including Africa [3]. The global battle against lymphatic filariasis (LF) is also challenged by the myths and superstitions prevalent among communities, people living with lymphatic filariasis (PLWLF), and healthcare providers [1,2].

The World Health Organisation launched the Global Programme to Eliminate Lymphatic Filariasis (GPELF) in 2000 to eradicate LF as a public health issue by 2030. The WHO envisaged that eradication could be achieved through strategies such as community distribution of mass drug administration (MDA) and the provision of essential care to alleviate suffering from LF. This initiative has successfully delivered more than 9 billion treatments to more than 935 million people in 70 countries. This has significantly curtailed the spread of LF, resulting in 17 countries eliminating it as a public health threat by 2021 [4,5].

As part of the strategies to alleviate suffering from LF, GPELF introduced a minimum package of care (MPC) for lymphoedema and LF, focusing on hygiene, wound care, and topical treatments, and encouraged the development of new diagnostic tools. In addition, GPELF encouraged the use of doxycycline and physiotherapy while cautioning against surgical interventions due to associated risks such as infection, nerve damage, lymphatic damage and worsening of the condition [6,7]. More recent research supports the safety of the use of a triple-drug regimen (ivermectin, diethylcarbamazine and albendazole) for LF, which is as safe as the double-drug approach [8]. In addition, the inclusion of mental health and rehabilitation services is promoted to improve the quality of life of affected individuals [9,10]

A study of Sri Lanka’s National Morbidity Management and Disability Prevention (MMDP) programme for PLWLF via SWOT analysis highlighted the need for better accessibility, coverage and resource optimization [11]. The Kerala model in India exemplifies the successful integration of lymphoedema treatment and self-management into public health systems—a scalable approach for LF-endemic regions that significantly enhances patient care access [12]. Other studies emphasize the necessity of integrating morbidity management and disability prevention and health education and prevention approaches into national health frameworks. This aids in addressing challenges such as stigma, resource limitations and the need for global support to meet GPELF’s objectives [13,14].

Studies in Africa underscore misconceptions around lymphatic filariasis and the importance of community engagement and healthcare provider training to improve service uptake [15]. Addressing these misconceptions would aid the rapid progress of LF elimination through integrated MDA, vector control and malaria control strategies [13,16].

Uganda’s national programme to eliminate lymphatic filariasis works towards managing and preventing disabilities from LF among high-risk individuals who are also found in malaria endemic communities, which is in line with international efforts. Despite significant strides, including the treatment of thousands of cases, challenges in changing community health beliefs and behaviours limit service effectiveness [17]. This reflects the difficulties in eradicating LF, emphasizing both progress and the ongoing need for targeted strategies, education, and global collaboration. There is an acknowledged gap in the understanding of the different factors and how they influence the success of morbidity management and disability prevention (MMDP) services, particularly in Uganda.

A situational analysis, site visits, and stakeholder workshops conducted earlier by some of the authors informed the need for this study and are detailed elsewhere [18]. The main findings revealed a multifaceted landscape of challenges and perceptions. The participants included but were not limited to PLWL, herbalists, and village health team (VHT) members, among others, and defined lymphoedema as swelling due to lymphatic blockage attributed to factors such as insect bites and genetic predisposition. Despite its integration into neglected tropical disease programs, there is no standardized case definition for lymphoedema that hinders effective management and referral within local health systems. Traditional herbalists are often sought for treatment, reflecting gaps in formal healthcare access and understanding. Myths associating lymphatic filariais with Bilharzia and witchcraft persist, influencing care-seeking behaviour. Social stigma, discrimination and inadequate healthcare infrastructure further complicate care delivery. MDA efforts face supply challenges and misconceptions, contributing to varied success across regions.

This study is underpinned by the socioecological model of health, which understands health from several intersecting levels and factors that cut across each other [19]. For example, the use of the model meant that we could explore how social, cultural, economic, and environmental factors, as well as policy, influence the prevalence, functional impact and risk factors associated with lymphatic filariasis in low-resource settings. By using this model, we can contextualise the broader societal and structural factors that contribute to the challenges faced in managing lymphatic filariasis effectively, ultimately contributing to health system strengthening.

This study seeks to better align policy and community engagement in lymphatic filariasis management, leveraging insights into community perceptions to refine interventions. In addition to supporting the objectives of the Global Programme to Eliminate Lymphatic Filariasis (GPEL), the management of people living with nonfilarial lymphoedema remains a significant public health concern in Uganda and beyond. This study aimed at exploring the barriers to accessing treatment for lymphatic filariasis through a socioecological lens in the Buvuma and Napak districts of Uganda.

## Methods

### Ethics approval and consent to participate

The Higher Degrees, Research and Ethics Committee (HDREC) at the School of Public Health (SPH-2023-508), College of Health Sciences, Makerere University Kampala, granted ethical clearance for the study. Furthermore, the study protocol received approval from the Uganda National Council for Science and Technology (HS3713ES). Permission to conduct the study was also obtained from the District Health Officers (DHOs) of the Buvuma and Napak districts. The participants who could read and write provided signed informed consent, whereas the remaining participants used thumbprints to provide consent before the commencement of the study. To improve the transcription process and protect the true identities of participants, identification numbers were assigned before the commencement of interviews.

### Study area and setting

Rural Uganda has particular challenges, and despite Uganda’s poverty alleviation programmes, many rural communities live in and in extreme poverty [20]. Buvuma and Napak districts of Uganda were randomly selected from six districts characterized by notably high poverty rates—where more than 40% of the population lives on less than $1.77 per person per day [21] and poor health coverage—where more than 45% of the population does not live within 5 km of a health centre, all of which contribute to challenges within their healthcare systems [22].

The six districts considered were Nabilatuk, Moroto, Pallisa, Buvuma, Napak and Amudat. Additionally, these two districts are in mosquito- and malaria-endemic areas, have some of the highest circulating filarial antigens, and are situated in two different geographic regions of the country—southern and northern [23,24].

Napak District comprises seven sub-counties and three town councils, with a population of 266,800 people. The district lacks a government hospital and a health centre IV, which are mandated by statutory regulations to have a qualified doctor. Instead, it has 18 health facilities, including six Health Centre IIs, 12 Health Centre IIIs, and one private hospital managed by Catholic Church Missionaries. Owing to this shortage, residents often must travel long distances to access affordable public health facilities. In the absence of public health facilities, privately owned drug shops serve as the primary point of ambulatory care for locals [25,26].

Buvuma District consists of 52 islands, three town councils, and six sub-counties, with a total population of 116,500 people. The district has one health centre IV, three health centre IIIs, and eight health centre IIs. Owing to its many islands, Buvuma District faces significant health system challenges, including inadequate health personnel and occasional drug stock-outs [27,28]. According to the Health Management Information System statistics from the Ministry of Health, in 2023, individuals affected by lymphatic filariasis in these districts did not seek care related to their condition at any health facility [29].

### Study design, participants and sampling procedures

A qualitative investigation utilizing in-depth interviews (IDIs) among people living with lymphoedema, key-informant interviews (KIIs) with health facilities in charges, district health officers (DHOs), and village health team members (VHTs), as well as focus group discussions (FGDs) with community members, was undertaken to obtain information following the standard practice of qualitative methods [30,31].

In addition to a desire to remain closely connected to the data, the researchers aimed at gaining insights into the knowledge that PLWLF possesses about the condition. This inquisitiveness extended to understanding the awareness of lymphoedema within the surrounding communities and among healthcare workers. Researchers have sought to explore the challenges of those affected by lymphoedema—specifically, where they seek treatment, the nature of the treatment received, the way health workers interact with them, and the actions taken by healthcare professionals when individuals with lymphoedema present themselves at health facilities. Finally, the investigators aimed to explore perspectives on what the government could do for people living with lymphoedema. This involved collecting viewpoints from PLWL, the communities in which they reside and the healthcare workers responsible for their management in health facilities. This comprehensive exploration led to the utilization of a qualitative inquiry method [32,33].

A purposive sample aimed at targeting individuals and communities where people with lymphoedema reside, which was crucial for our research question to be answered, was recruited. The study included eight focus group discussions in Napak district and 11 in Buvuma district. Additionally, seven KIIs were conducted in Napak and five in Buvuma district, along with nine in-depth interviews in Napak district and 12 in Buvuma district. The recruitment process involved the researchers reaching out to participants through the District Health Officer during a previsit conducted before data collection. The assistance of the District Health Officer (DHO) was sought to facilitate participant recruitment because DHOs typically have an extensive network within the local community, making them well positioned to identify potential participants who meet the study criteria. We leveraged their connections to streamline the recruitment process and ensured that the research teams reached individuals who were relevant to the study. Moreover, participants were more willing to participate in the research when approached by a person sent from the DHO office.

The investigators wrote letters of intent to conduct the study, which were submitted to the District Health Officers of the Buvuma and Napak districts. These letters were accompanied by ethical clearance obtained from the Makerere University School of Public Health (MakSPH) Research and Ethics Committee. The District Health Officers then engaged the district tuberculosis and leprosy focal persons, who also serve as focal persons for neglected tropical diseases. These focal persons, in turn, contacted community health workers, also known as village health teams in Uganda, to assist in identifying individuals with lymphoedema for in-depth interviews and community members for focus group discussions.

A total of 152 community members participated in nineteen focus group discussions, with each group comprising 7 to 9 participants, as is prescribed for FGDs [34]. Additionally, individuals with superior knowledge of lymphoedema participated in 12 key informant interviews, while 21 participants were involved in in-depth interviews. In the Buvuma district, two men from different villages dropped out of the FGDs due to fishing commitments, and in the Napak district, three women left ongoing FGDs to fetch firewood.

### Data collection

Undertaking fieldwork in these districts presented many challenges for researchers. For example, in Buvuma Islands, the study team had to hire a speed boat to access some of the Islands that were one and a half hours away from the main Island. When the weather conditions were not good, such as when it rained, data collection had to be halted until the following day or until the weather was good enough to travel by boat. In Napak District, the study team had to move between 9 am and 4 pm to avoid being caught up in any security situation that may have arisen because of cattle rustling by the indigenous Karamojong. This also slowed the data collection process.

The interview guides used for data collection were designed by the research team in English but were translated into different local dialects by the Makerere University School of Languages. The guides, designed with prompts and thoroughly reviewed by the research team, were utilized to collect information from community members. Some of the key questions asked of the participants included the following: has lymphoedema affected your ability to go to work or school? What are the cultural beliefs and attitudes about lymphoedema in Uganda? What resources or support have been helpful to you? Do you face any stigma and discrimination due to your condition? Where do you receive treatment from and what treatment do you use to manage lymphoedema?

The interview guides were developed in reference to literature from the Lymphoedema Impact and Prevalence International (LIMPRINT) study on quality of life and were supported by an international team of Lymphoedema and methodology experts [35]. Questions embedded in the guides, such as where treatment is commonly sought from by people living with lymphoedema, were also added to the guides, as this has been documented to improve Universal Health Coverage (UHC) [36]. FGDs were held with small groups of people within the community who were asked about their perceptions, opinions, beliefs and views regarding lymphoedema [37]. In both districts, FGDs took place beneath trees situated in the respective villages, providing a secluded environment away from the broader community. Relatedly, key informant and IDI guides were used to gather information from people who were more knowledgeable about and those living with lymphoedema, respectively [38]. IDIs were carried out within the homes of PLWL, whereas KIIs were conducted in various offices corresponding to the interviewees. In instances such as where the key informant was able to speak English, the interviews were conducted in English. Otherwise, selected research assistants fluent both in English and the local languages were trained and deployed for the task. In the Buvuma district, we selected research assistants fluent in English, *Lusoga* and *Luganda*, whereas in the Napaka district, we chose research assistants fluent in English and *Karimajong*.

Throughout all the instances, the interview settings were carefully chosen to be free from noise disturbances. The interviews exclusively involved the researchers and participants maintaining a private and focused atmosphere. Notably, no additional demographic information was gathered from the participants during the interviews. The study team also wanted to understand how the costs of accessing treatment impact PLWL. As such, questions from studies looking at a similar attribute were added [39]. All the interview guides underwent pilot testing in Kampala, the capital city of Uganda, to ensure their effectiveness in collecting the intended information. In the Buvuma district, interviews and focus groups were conducted by one researcher (AB), who was assisted by a moderator. Moreover, in Napak district, the interviews were carried out by a researcher (GO), and the focus groups were conducted by another researcher (AL), who was assisted by one moderator each.

At the time of the study, AB held a PhD and was a research fellow, whereas GO and AL possessed master’s degrees and were research assistants involved in various research projects. All three researchers—AB, GO and AL—were male. AB, equipped with prior training and experience in qualitative research methodologies, conducted interviews and focus groups in the Buvuma district. In contrast, GO and AL underwent comprehensive training in qualitative research methodologies before embarking on fieldwork for data collection in Napak district.

The researchers and participants had no preexisting connections. During the introduction of the study to the participants, information about the researchers’ affiliations and the research’s purpose were mentioned. The three researchers expressed interest in the study, motivated by the fact that lymphoedema is considered a neglected tropical disease in Uganda, with minimal prior research conducted in this domain.

All interviews and focus group sessions were audio-recorded, but field notes were not generated during or after the interviews. Field notes were not taken, as they provided less distraction and interruption, as we wanted to ensure that the participants felt comfortable and free to express themselves without interruptions or distractions. This enabled the researchers to capture authentic responses and insights without imposing external influences or biases that could arise from the notetaking process. The duration of FGDs ranged from 45–65 minutes, whereas the durations of KIIs and IDIs varied between 30–55 minutes. Data saturation—the point at which no new information emerged—was observed in different stages. [40]. For FGDs, saturation was reached by the seventh and eighth sessions in the Napak and Buvuma districts, respectively. In the case of the KIIs, saturation occurred in the fourth and sixth interviews in the Buvuma and Napak districts, respectively. Saturation for IDIs was reached by the eighth interview in both districts. Additional interviews were conducted to ensure that no other issues arose after saturation. The research team did not conduct repeat interview sessions in the study districts, and transcripts were not returned to participants for comments because they were transcribed in English, which was unfamiliar to most interviewees and made it difficult to access the study participants.

### Data management and analysis

All audio recordings were transcribed verbatim into Microsoft Word documents, and the transcribed scripts were subsequently verified through simultaneous reading and listening to the audio files. The transcribed scripts were uploaded into Atlas version 7 for coding and subsequent analysis.

Initially, one FGD, one KII, and one IDI were independently coded by AB and DM. Following this, a discussion was conducted to achieve agreement and consensus on the developed codes as well as to address any differences that may have arisen. This process aimed at enhancing the validity and reliability of the developed codes. Subsequent transcripts were coded using the agreed-upon codes. The same procedure of discussion and consensus-seeking was employed for subcategories, categories and themes to ensure the rigor of the study. In both scenarios, discussions continued until thematic saturation was attained—meaning discussions and consensus-building persisted until new codes and themes no longer added new insights beyond the existing ones.

The final thematic analysis adhered to Graneheim and Lundman’s framework [41] for capturing both latent and manifest content. Codes with similar or closely related meanings were identified and collated to form subcategories. These subcategories were further condensed to create categories. The final analysis resulted in a larger narrative of themes derived from condensed categories. Following the analysis, a draft report was prepared, and a dissemination meeting was conducted with the District Health Officer (DHO) and District TBLP (Tuberculosis and Leprosy Program) focal persons and community members in the Buvuma and Napak districts, respectively.

A debrief session was also held with the key informants to validate the study findings and address issues of rigor and trustworthiness in the collected qualitative data. During the dissemination meetings, participants were invited to provide feedback on whether the study findings accurately reflected the content of the interviews conducted at the time of data collection.

## Results

The study findings are presented under three main themes: stigma and social isolation, health care disparities, and health care dynamics. These themes are cut across four levels of the socioecological framework—individual, community, organizational, policy and structural/environmental.

### Stigma and social isolation

PLWLF face significant emotional and mental distress due to stigma and isolation. The interviews revealed that community beliefs contributed to feelings of neglect and exclusion. The stigma often results in difficulties in engaging in social activities, resulting in individuals with lymphoedema withdrawing and self-isolating. Even at the community level, stigmatization and isolation were prevalent since communities in both districts had inadequate awareness of hydrocele and lymphatic filariasis, leading to social isolation. For example, in Napak district, there was a universal belief in the curse associated with conditions such as hydrocele, which affects community integration and support for affected individuals. In most of the focus groups conducted among females in Napak district, there was a consensus that there was psychological impact and societal withdrawal due to community beliefs:

*“People with ‘lobukejen’ [lymphatic filariasis] fear living among other people in the community. They cannot walk under the hot sunshine; they become lazy and fail to work due to body weakness.”* Respondent 7, FGD females, Nakorete, Napak

In both Napak and Buvuma districts, the beliefs held by individuals and the community led to social stigma and self-isolation because of the way communities understood the causes of conditions such as lymphatic filariasis. Consequently, these beliefs significantly impact how individuals manage these illnesses and seek treatment. The study revealed that strong cultural beliefs, particularly PLWL, played a crucial role in shaping their perception of illness and guiding their coping strategies and approaches to seeking medical assistance.

*“During the time of our forefathers, there used not to be such a disease as Lobukejen [lymphatic Filariasis]; however, there was a common disease that could cause stomach and feet to swell but could easily be treated with traditional herbs, and they could respond effectively. However, the people have also tried to do the same for Lobukejen, but it is not responding. Instead, it becomes worse. There is misconception about the disease, where people believe that swollen feet are not caused by Lobukejen but are caused by witchcraft, especially if someone has stolen from somewhere.”* Respondent 6, FGD Females, Nakichmet, Napak

Among the people who were more knowledgeable about lymphoedema and community members in both districts, most of the people with lymphatic filariasis and hydrocele did not access health care since they felt that healthcare providers did not have a solution; they would be stigmatized on the way to the health facilities and, as such, had lost hope. Often, people with lymphoedema feel isolated and discriminated against. The following quotes were obtained from the study participants:

*“People have lost hope either from the government or traditional healers for the cure of lymphoedema. The only hope they have is in God. Through prayers, they believe they will get healed from the disease.”* Respondent 3, FGD Females, Nakichumet, Napak

and

*“For health workers*, *discrimination is prevalent*, *primarily due to stigma*. *When they [people with lymphoedema] go to fetch water*, *people do not want them to step into the water*, *fearing it will become contaminated and they will get infected as well*.*”* Health Worker, Napak Town Council, Napak

The interviews also revealed varying impacts across the different levels of society. Individuals with hydrocele or lymphatic filariasis experience significant psychosocial impacts, including feelings of isolation, depression, and hopelessness, because of the physical and visible nature of their condition. As a result, individuals feel stigmatized, have negative perceptions, and have reduced self-esteem, leading to social withdrawal. The majority of PLWL interviewees reported the following:

*“Yes, many times I live in denial, feel lonely, self-neglect and self-pity especially the time when the weather is so cold, and the pain is extreme and hard to bear.”* Indepth Interviewee, Male, Lyabaana, Buvuma

While the key informant said:

*“Some patients with hydrocele are devastated with life and feel neglected because of their condition*, *but there are few women who are committed to and standby their husbands*. *However*, *we don’t ask them much*, *unless when they open up to us*, *we normally counsel them for only those who confide in us and advise them to go for surgery*.*”* Nurse, Busamuzi, Health Center III, Buvuma

Within the communities of Buvuma and Napak, there was profound stigma associated with visible and misunderstood health conditions. The stigma led to social ostracization of the individuals affected by the condition to the extent that they were excluded from social gatherings and community activities. The communities also harboured misconceptions about the contagiousness of the condition, leading to further discrimination and isolation of the affected individuals.

*“They face stigma from the community. They are always isolated by the people because they believe that it is contagious. They are always humiliated and demoralized. For example, if there are ceremonies at home, they are isolated. You find that they face many challenges from the community in which they are living. Some of them even hang themselves because they are demoralized. Then, economically, they are not able to do much work because of the condition.”* Health workers, Nakorete, Napak

### Healthcare disparities

While conducting the study, healthcare facilities struggled to provide adequate care for PLWL because they did not have access to resources to manage the condition. This was aggravated by the lack of trained healthcare personnel. These challenges are enhanced by ineffective government and institutional policies and a lack of training for healthcare personnel from medical schools with inadequate knowledge of lymphoedema.

*“We are asking the government to give us medicine and, if possible, pay for the operations because most of us cannot afford it. Additionally, they should expand our health facilities here so we can easily find help, especially for the children.”* Respondent 5, FGD Males, Kijjo, Buvuma

The key informants highlighted the profound impact of the inadequate health care available, which contributes to the mental health challenges of PLWL, illustrating the despair and lack of hope that they often feel, which can lead to severe social and psychological consequences.

*“Yes, the one I have encountered was a patient with lymphoedema. The person had lost hope and, most of them are not mentally well, and the one I saw had resorted to street begging because they feel they have lost hope and feel nothing can be done for them.”* Key informant, Health Center IV, Buvuma

Inadequate health system challenges were also reflected in the way community members understood the causes of lymphoedema. Most community participants seemed not to be sensitized by competent health care personnel and, as such, attributed lymphoedema to be due to supernatural causes impacting the way communities interact with people living with the condition. In most of the key informant interviews conducted in Napak, conditions such as lymphoedema are seen as either curses from ancestors, supernatural forces, or consequences of criminal actions.

*“People here in Karamoja have different ideologies. Some think it is a curse from the elders who could have abused them, whereas others believe it is a disease that runs through a specific clan or family. However, the main cause is believed to be a curse from ancestors or when they go and raid an area with these people living with LF and they kill them, it is believed that this condition follows the criminals.”* Key informant, health worker, Napak

Relatedly, healthcare providers in these two districts believe that they face challenges due to deeply entrenched community beliefs that hinder effective medical interventions where they exist. As a result, in both districts, there was a prevalent reliance on traditional healers and herbal remedies driven by the belief in witchcraft as the cause of illness. This belief significantly undermines trust in conventional medical treatment, complicating the efforts of healthcare professionals to provide modern medical care.

*“We have herbalists who get the herbs from forests and come here in this village to deceive people who the herbs will treat hydrocele, but all I know these just want to extract money from people, but the herbs they give people cannot treat the hydrocele.”* Indepth Interviewee, Lyabaana, Buvuma

Using traditional medicine when there is no other solution might make sense to the sufferer well. Evident from the FGDs was the fact that the government and policymakers need to address causal beliefs by enhancing health education and health literacy. The need for continuous sensitization to the actual causes of the disease and debunking myths is essential for improving health outcomes.

*“The government should have continuous sensitization of the disease [lymphoedema], especially with respect to its causes, and to help demystify some myths on the causes of the disease. The government should also give mosquito nets to the people so that the people don’t get the disease from mosquitoes.”* Respondent 2, FGD Males, Lomuria, Napak

At the organisational level, healthcare facilities in both districts were underequipped and lacked the necessary resources to provide adequate treatment. Healthcare workers in both districts reported using what was available, such as diclofenac injections for pain relief and antibiotics for infections, both of which are appropriate but were unable to receive any other specialized treatment since they were unavailable. One health worker noted:

*“For those hydrocele patients who come to the facility with pain, we manage the pain using a diclofenac injection. Sometimes, we give them antibiotics and pain killers such as flagil, amoxyl, antibiotics and panadol because those are the medications we have at the facility.”* Nurse, Health Center III, Buvuma

From a policy standpoint, there is a notable disparity between the cultural perceptions within communities and the scientific comprehension of the illness, which hinders access to health care. While certain members of the community believed that hydrocele (a medical condition involving swelling of the scrotum) could develop because of respect or high status historically linked to having the condition, others attributed it to witchcraft, despite also regarding it as a prestigious affliction.

*“I grew up being told that people would buy hydrocele. Our ancestors thought it was very prestigious to be seen with it or known to have it. However, right now it is a disease, and I don’t know how it is caused.”* Respondent 2, FGD Females, Tojjwe, Buvuma

### Health care dynamics

Structurally, both districts presented notable disparities in healthcare infrastructure and access. The geographical remoteness of health facilities coupled with deep-rooted traditional beliefs presented substantial obstacles to accessing and delivering medical care. Moreover, the dependence on intermittent medical interventions due to the absence of permanent healthcare facilities further worsened these challenges. One of the participants said:

*“We usually go to hospitals, especially those in Jinja, Kampala, Mulago and Iganga, where they carry out the operations and treat LF. Here on the Island, we don’t have doctors that can treat us.” Respondent 5, FGD Males, Lukaale, Buvuma and another said “Sometimes here, there are diseases that are complicated and cannot be managed or treated by medical personnel in hospitals*. *Therefore*, *patients go to seek help from other sources such as herbalists*.*”* Key informant, Religious leader, Napak

It was clear from most of the FGDs that in both districts, environmental and structural aspects, such as living conditions and local ecosystems, are often intertwined with local beliefs about disease causation, which affects health-seeking behaviour. There is a widespread belief that water and associated parasites cause conditions such as lymphoedema, which influences both the perceived risk and the preventive measures the community is willing to engage in.

“*Sometimes, it is like this condition is also got from stepping in dirty water. When a child is born, the legs start swelling, and you wonder where this problem has come from. Is it through sexual intercourse or, is it from our movement in the bushes, so it is truly confusing. We know that there is no hospital to rescue us now.*” Respondent 6, FGD Males, Lokiteded, Napak

and

*“I hear people say that lymphoedema is caused by parasites*. *One person acquires it after stepping in water*, *while others acquire it after stepping in a garden of a person who practises witchcraft and that person bewitches his/her feet*. *We are told that the hydrocele is just swelling*. *We know the condition but don’t understand it*.*”* Respondent 6, FGD Females, Lukaale, Buvuma

Environmental factors such as the geographical spread of diseases linked to water bodies and the lifestyles of communities (such as fishermen in Buvuma and cattle keeping in Napak faced increased risks) play a significant role in the management of hydrocele in both districts. The following quotes reflect the significant role that environmental factors play in the management of the conditions in the Buvuma and Napak districts.

*“Given the nature of my job, like I had informed you earlier, staying in water all night and day fishing, brought me the hydrocele”.* Indepth Interviewee, Kembo, Buvuma

*“People get lymphoedema from dirty and contaminated water bodies; when a fly bites someone from the water*, *it infects them with the disease*. *Most of the people who moved to teso*, *Jinja*, *Mbale and Kenya where rivers and lakes exist and if they stepped in those water bodies*, *they returned infected with the disease”*. Respondent 3, FGD Females, Nakichumet, Napak

**Fig 1:**
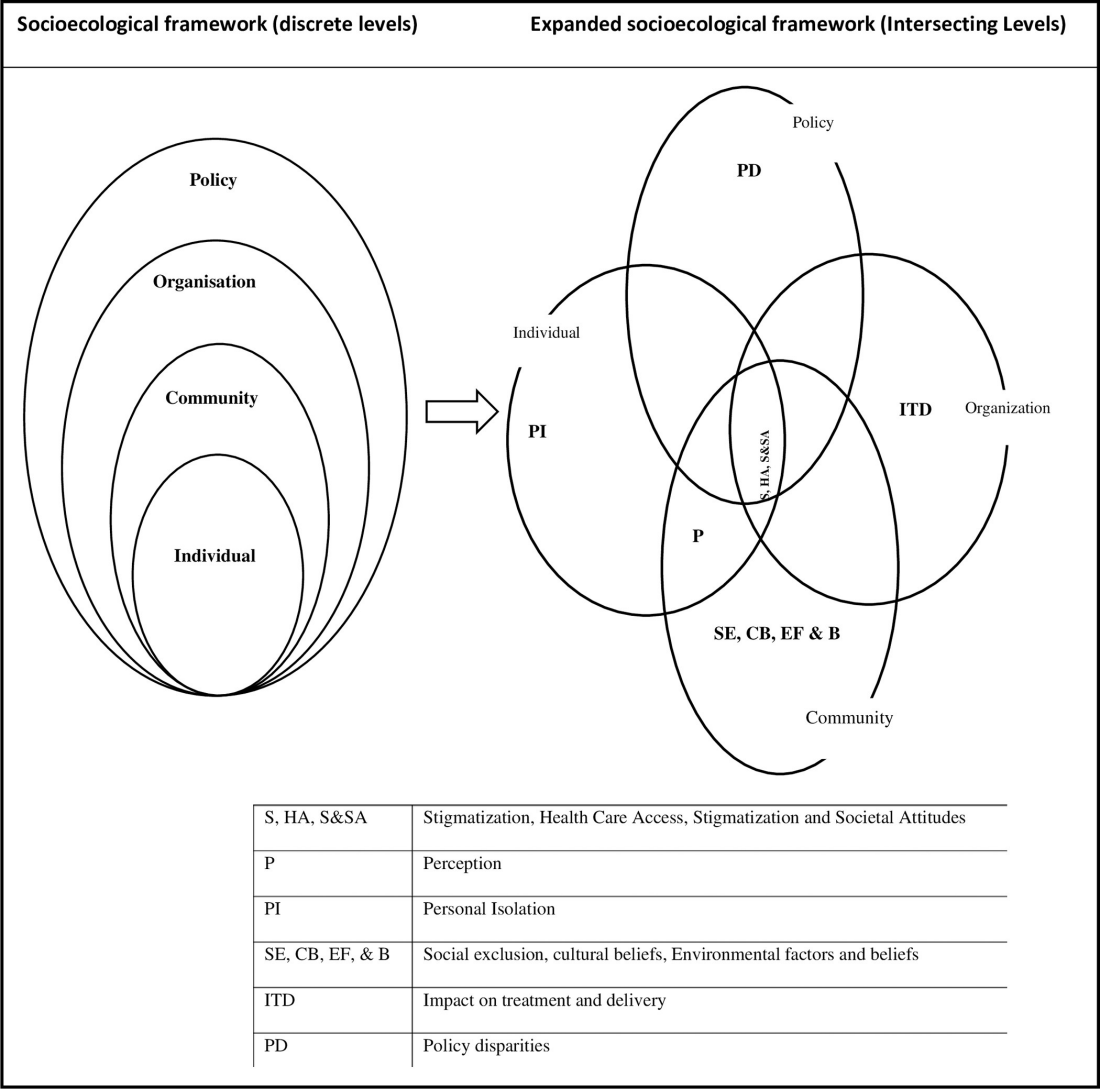
Expanded illustration of the socioecological framework showing intersections with various levels.

The study revealed a complex network of interconnected factors, as depicted in the expanded socioecological framework in Fig 1.

This diagram illustrates how various elements impact the experience of PLWL in the Buvuma and Napak districts of Uganda. Importantly, the barriers within each level of the socioecological framework are not isolated entities. While personal isolation, policy disparities, treatment impacts, and delivery issues, along with a blend of poverty, social exclusion, cultural beliefs, and environmental factors, may seem to be distinct barriers, the study findings suggest that they are interwoven and mutually constructed.

## Discussion

This study aimed at exploring the challenges and factors associated with a lack of access to treatment among PLWLF in the Buvuma and Napak districts of Uganda. This study was premised on the fact that the WHO and Ministry of Health-Uganda have endeavoured to curtail interpersonal transmission of the disease through various strategies. Nonetheless, morbidity control remains difficult, especially among those living with this condition. Overall, three themes emerged from the study: stigma and social isolation, health care disparities, and health care dynamics.

By exploring individual experiences and coping mechanisms, this study provides insights into the realities faced by PLWLF. By highlighting community beliefs and attitudes, the study sheds light on the social context within which affected individuals navigate their health conditions. By examining healthcare infrastructure and policies, this study identifies systemic barriers to access and treatment. By considering broader societal attitudes and environmental conditions, this study highlights the challenges faced by individuals and communities affected by lymphoedema and LF.

The study results show that PLWLF are stigmatized and socially isolated by community members because of their conditions. This lowers self-esteem in those with this condition and makes it difficult to find work where it exists that can potentially elevate their socioeconomic status. These results are similar to those of other authors, who reported that PLWL individuals experience significant stigma within their communities, including feelings of social isolation [42–44]. These studies emphasize the psychological effects and economic impacts experienced by individuals with lymphoedema, shedding light on the challenges they face owing to societal attitudes and misconceptions and a lack of understanding of the causes of lymphatic filariasis. There is also a need for health systems to implement stigma reduction interventions aimed at addressing the social stigma and discrimination faced by PLWL through addressing the existing knowledge gap. This may involve community sensitization programmes, health education campaigns, and advocacy efforts to challenge the negative attitudes and perceptions held by community members towards the condition.

In addition to stigma and social isolation, PLWLF were unable to access treatment because of poorly resourced health systems in both districts. The lack of adequate and trained medical personnel as well as appropriate medical equipment was mentioned. Even when some form of treatment is used, it does not yield the desired outcomes. For the very few people who had undergone surgery for hydrocele, there was much scepticism surrounding the recurrence of the condition. Even those who went to traditional healers did not seem to see any tangible reduction in the size of the swelling, despite surgery being an approved intervention for hydrocele [45]. The study results also revealed that the few people who had undergone hydrocele surgery faced recurrence, as has happened elsewhere, highlighting the need for epididymis resection during hydrocelectomy [46]. Therefore, there is a need for health systems to prioritize initiatives aimed at enhancing effective management of lymphatic filariasis. This should involve taking a health promotion approach and training healthcare providers and community health workers in cost-effectiveness, self-management techniques, low-cost and early intervention solutions at the community level. Having access to more specialized treatments and medical interventions, developing protocols for postoperative management, including follow-up assessments, monitoring for complications and providing rehabilitation support, are long-term initiatives that need to be planned.

For most of the study participants, poverty, lack of decent work and lack of access to resources were the main reasons why they could not access adequate management for lymphatic filariasis. This is in tandem with studies that have highlighted the fact that poverty exacerbates health disparities and is a significant predictor of increased infection risk, adverse health outcomes, and higher mortality rates [47,48]. While the aforementioned may be true on the PLWL side, this is further complicated by the fact that health facility management of lymphoedema may also include the use of complex decongestive therapy (CDT) and good skin care—depending on the stage of lymphoedema—all of which may be costly for many PLWLF in LMIC countries. Specialized management techniques require highly specialized personnel who are not readily available in many LMICs [49]. Therefore, these health systems face significant challenges in providing adequate care for LF, necessitating strategies that address both financial and human resource constraints.

The geographical remoteness of the districts coupled with distant health facilities for those who live with lymphatic filariasis was considered a major healthcare challenge affecting the management of the condition. Several studies have pointed out that geographical remoteness is a significant barrier to health care access, especially for vulnerable and poor people, similar to the results of our study [50,51]. Health systems need to allocate more resources to improve infrastructure, such as transportation and telemedicine services, to bridge the gap between patients and healthcare providers. There is also a need to deploy more healthcare professionals in remote areas and provide incentives for them to work in underserved regions. The development of community-based health programmes and training local health workers can help provide basic care and support for PLWLF, reducing the need for long-distance travel to health facilities.

The findings of the study also show that in the study districts, a range of beliefs regarding witchcraft’s role in the causation of lymphatic filariasis exist. In addition to being affected, other causes of lymphoedema such as congenital lymphoedema, contact with contaminated water and insects were mentioned in Zambia, where PLWL alluded to witchcraft as a cause of the condition [43]. However, while the study participants in Uganda mentioned contaminated water and insects as possible additional causes of the condition, in Zambia, the participants mentioned contact with animal faeces and/or the use of aphrodisiacs as perceived causes of LF. These results highlight the cultural complexities surrounding health and illness in these communities. Understanding the beliefs and perceptions surrounding LF is crucial for effective health education campaigns, as this understanding will address the existing misconceptions about this condition. Health interventions should be tailored to address the specific beliefs and perceptions of communities in different regions.

Finally, undertaking research in such challenging environments requires careful planning for fieldwork because an unforeseen extended duration of fieldwork could increase costs and strain resources. In addition, coordinating travel and ensuring the safety of the research team and equipment in such difficult conditions is paramount and might require additional measures and protocols.

### Study strengths and limitations

The major strength of this study was that we used different methods of data collection (focus group discussions, key informant interviews and in-depth interviews), which enabled the triangulation of findings. By using a combination of these qualitative research methods, we were able to gather comprehensive data, observe group dynamics and delve into individual experiences. Each method was able to offer a unique perspective and depth of understanding and contribute to a holistic view of the challenges of access to treatment for PLWLF. The novelty of this study lies in its comprehensive application of the socioecological model (SEM) to understand the various dynamics influencing the utilization of healthcare services and the management of LF in the Napak and Buvuma districts. The study relied on qualitative methods, including focus group discussions, key informant interviews and in-depth interviews. While these methods allow for a comprehensive understanding and triangulation of data, the findings are limited to the perspectives and experiences of the study participants and may not be generalizable to all PLWL in Uganda or other settings.

## Conclusion

This study provides valuable insights into the reality that while tremendous efforts to curtail interpersonal transmission have been made by different stakeholders, morbidity management in Uganda remains largely unaddressed. Among PLWLF in Uganda, there is a need to address sociocultural beliefs, social and interpersonal dynamics, health care disparities and dynamics if patient outcomes and well-being are to improve. This information will aid in the overall management of lymphoedema in Uganda and other similar settings.
